# Psychological

**Published:** 1848-03

**Authors:** 


*Psychological.*—During an attack of delirium, many people have learned to read and write with great rapidity, but have been unable to do either after their reason returned, and increased determination of blood to the brain had ceased. Another attack of insanity, however, revived their memory and their ability to read and write. Many people have their recollection of past events wonderfully restored by dreams. Several instances of this kind are related by Dr. Abercrombie, in his "Inquiries concerning the Intellectual Powers ;" but they may be accounted for by the increased activity of certain portions of the brain, during sleep. In somnambulism, which differs but little from dreaming, some persons have been able to recollect things long forgotten, and to talk in a language of which they possessed no knowledge when awake, but with which they had in early life some partial acquaintance. This wonderful power of the memory has been frequently exhibited, by a few, when under great excitement; and, in ignorant and fanatical times, has induced a belief in the gift of tongues. Those who had learned but little of a language when young, and had totally forgotten it, were now, when in a convulsive state, able to speak it fluently; while others were able to repeat long passages from books that they had never read but once, and had not seen for many years. Similar effects have been produced by *animal magnetism*, which, as every one knows, powerfully affects the imagination. During the state of "extase," caused by *magnetism*, the memory has often been surprisingly perfected; and some have been rendered able to speak in a language they had long forgotton. This state was always accompanied by symptoms that showed an increased determination of blood to the head. All had slight convulsions, the face became red, the eyes bright, and after awhile humid.

Like effects are produced by disease of the brain, which sometimes will revive whatever has been at any time in the memory. They are not rare, says M. Bertrand, in all diseases which greatly excite the brain. M. Moreau (de la Sarthe) says, in the *Encyclopedie Methodique*, (Art. Medicine Mentale,) that he had the care of a child twelve or thirteen years of age, who had studied the Latin language a little, but was not able to speak it when in health, but who, during the excitement of fever, spoke it fluently.

The following case which occurred in Germany, is related in Coleridge's *Biographia Literaria*:

A young woman of four or five-and-twenty, who could neither read nor write, was seized with a nervous fever; during which, according to the asseverations of all the priests and monks of the neighborhood, she becamed *possessed*, and, as it appeared, by a very learned devil. She continued incessantly talking Latin, Greek, and Hebrew in very pompous tones, and with most distinct enunciation. The case had attracted the particular attention of a young physician, and, by his statement, many eminent physiologists and psychologists visited the town, and cross-examined this singular case on the spot. Sheets full of her ravings were taken down from her own mouth, and were found to consist of sentences coherent and intelligible, each for itself, but with little or no connection with each other. Of the Hebrew, a small proportion only could be traced to the Bible; the remainder seemed to be Rabbinical dialect. All trick or conspiracy was out of the question. Not only had the young woman ever been a harmless, simple creature, but she was evidently laboring under a nervous fever. In the town in which she had been residing for so many years, as a servant in different families, no solution presented itself. The young physician, however, determined to trace her past life, step by step; for the patient herself was incapable of returning a rational answer.

He at length succeeded in discovering the place where her parents had lived; travelled thither, found *them* dead, but an uncle surviving; and from him learned that the patient had been charitably taken by an old protestant pastor at nine years, and had remained with him some years, even till the old man's death. Of this pastor the uncle knew nothing, but that he was a very good man. With great difficulty, and after much search, our young medical philosopher discovered a niece of the pastor's, who had lived with him as house-keeper, and had inherited his effects. She remembered the girl; related that her venerable uncle had been too indulgent, and could not bear to have the girl scolded; that she was willing to have kept her, but that after her patron's death, the girl herself refused to stay. Anxious inquiries were then, of course, made concerning the pastor's habits, and the solution of the phenomenon was soon obtained. For it appeared, that it had been the old man's custom for years, to walk up and down a passage of his house, into which the kitchen door opened, and read to himself, with a loud voice, out of his favorite books. A considerable number of these were still in the niece's possession. She added, that he was a learned man, and a great Hebraist. Among the books were found a collection of Rabbinical writings, together with several of the Greek and Latin Fathers; and the physicians succeeded in identifying so many of the passages with those taken down at the young woman's bed-side, that no doubt could remain in any rational mind, concerning the true origin of the impressions made on her nervous system.

This authentical case furnishes both proof and instance, that relics of sensation may exist, for an indefinite time, in a latent state, in the very same order in which they were originally impressed; and as we cannot rationally suppose the feverish state of the brain to act in any other way than as a stimulus, this fact, (and it would not be difficult to adduce several of the same kind,) contributes to make it even probable, that all thoughts are, in themselves, imperishable; and that if the intelligent faculty should be rendered more comprehensive, it would require only a different and apportioned organization, *the body celestial* instead of *the body terrestrial*, to bring before every human soul the collective experience of its whole past existence. And this—this, perchance, is the dread book of judgment, in whose mysterious hieroglyphics every idle word is recorded! Yea, in the very nature of a living spirit, it may be more possible that heaven and earth should pass away, than that a single act or a single thought, should be lost.—*American Journal of Insanity.*

